# Comparison of Antioxidant Properties and Color of Selected Polish Honeys and Manuka Honey

**DOI:** 10.3390/foods13172666

**Published:** 2024-08-24

**Authors:** Ewa Majewska, Beata Drużyńska, Dorota Derewiaka, Marta Ciecierska, Paulina Pakosz

**Affiliations:** Division of Food Quality Assessment, Department of Food Technology and Assessment, Institute of Food Science, Warsaw University of Life Sciences, Nowoursynowska 159 Street, 02-787 Warsaw, Poland; beata_druzynska@sggw.edu.pl (B.D.); dorota_derewiaka@sggw.edu.pl (D.D.); marta_ciecierska@sggw.edu.pl (M.C.); paulina_pakosz@sggw.edu.pl (P.P.)

**Keywords:** honey, Polish honey, manuka honey, antioxidant activity, polyphenols, color

## Abstract

The antioxidant capacity and nutritional value of honey are significantly dependent on the content of phenolic compounds. The aim of this study was to compare the antioxidant properties and color of selected honeys and manuka honeys available in the Polish market. The results showed quantitative differences in phenolic acids, phenolic content and antioxidant activity between the honeys, indicating the influence of floral sources. Dark honeys, including buckwheat honey, showed increased phenolic content and superior antioxidant properties. The study revealed remarkable correlations between phenolic content, antioxidant capacity and color. Buckwheat honey showed higher antioxidant properties compared to manuka honey, which is highly valued in the current market. These results highlight the importance of further research into Polish buckwheat honey and advocate its wider consumption due to its high nutritional value and remarkable bioactive properties. In addition, the study contributes to a deeper understanding of honey diversity and highlights the potential importance of regional honey varieties in promoting health.

## 1. Introduction

According to legal regulations [1], honey is defined as a natural sweet substance produced by bees (*Apis mellifera*). It originates from plant nectar or from secretions of living plant parts or excretions of plant-sucking insects on living plant parts. Bees collect, transform and process this substance, incorporating their specific ingredients into it. The resulting mixture is deposited, dehydrated, stored and left to ripen and mature in honeycombs.

Honey can be considered a concentrated solution of sugars, consisting of fructose, glucose, sucrose and maltose. It also contains proteins, enzymes (e.g., catalase), amino acids, minerals and vitamins [2,3,4,5]. Honey is also rich in polyphenols and phenolic acids, which are secondary plant metabolites. Studies have shown that the content of these substances varies between different plant sources, resulting in significant differences in the composition and content of phenolic compounds in different monofloral honeys. Phenolic compounds are recognized as the primary constituents responsible for the health-promoting properties of honey, e.g., anti-microbial, anti-inflammatory, anti-mutagenic, anti-tumor, antiviral, antioxidant capacity and many other effects on human health [6,7,8]. The therapeutic potential of honey is closely related to its antioxidant activity, and it has been established as a well-known natural antioxidant [6,9]. Despite the widespread use of antibiotic and chemotherapeutic compounds, the role of honey as an anti-microbial agent remains significant [10].

Honey’s color is the most important sensory factor for consumers. This characteristic is predominantly dependent on the honey’s nectar source and pollen content [11]. Changes in honey color can occur slowly during storage or rapidly during thermal treatment [2]. Several studies have shown that dark honeys exhibit higher levels of bioactive compounds compared to light honeys [7,12]. Consequently, the scientific literature confirms that dark honeys have better antioxidant activity than their light equivalents [3,6,11].

Polish honeys are valued as high-quality products, characterized by their rich composition of various compounds that significantly influence their properties. The Polish market offers a wide range of native honeys, including lime, rape, dandelion, heather, acacia, phacelia and goldenrod. The differences between these varieties are due to variations in the composition of polyphenols and other bioactive compounds [13]. In the age of globalization, more and more honeys from other climatic zones are appearing in the Polish market. One of these honeys is manuka honey, which has a high price due to its unique properties. Manuka honey is a monofloral honey obtained from the manuka myrtle tree (*Leptospermum scoparium*), which grows mainly in New Zealand [14,15]. Manuka honey is well known for its health properties due to its unique chemical composition. Manuka honey is characterized by its dark color [8]. There are studies in the literature comparing Polish honeys with manuka honey, but, for example, Kuś et al. [16] focused on the activity of Polish unifloral honeys against pathogenic bacteria and its correlation with color, phenolic content, antioxidant capacity and other parameters. Puścion-Jakubik et al. [17] investigated whether selected phenolic acids can be used as markers for individual varieties of Polish honeys and correlated the content of these acids with selected parameters determining honey quality.

The aim of this study was to compare the antioxidant properties and color of selected Polish honeys with manuka honey. Such a comparison can contribute to a better use of local honey resources, increase consumer awareness and promote the sustainable development of regional beekeeping.

## 2. Materials and Methods

### 2.1. Materials

In 2020, commercial honeys sourced from distinct floral origins were purchased in various markets and stores. The floral sources and corresponding codes are as follows: acacia (*Robinia pseudoacacia*)—H1, phacelia (*Phacelia tanacetifolia* Benth.)—H2, lime (*Tilia*)—H3, rape (*Brassica napus*)—H4, multifloral—H5, buckwheat (*Fagopyrum esculetum*)—H6, honeydew—H7 and manuka MGO-250 (*Leptospermum scoparium*)—H8. All samples were kept in the dark at room temperature until they underwent processing. This standardized storage protocol ensures the preservation of the honeys’ original characteristics for subsequent analyses.

### 2.2. Methods

All analyses were performed in triplicate.

#### 2.2.1. Water Content

The water content (moisture) was determined based on the standardized refractometric method proposed by the IHC [18] using an Abbe-type refractometer (PZO, Warsaw, Poland). Using the Wedmore table, the percentage of water content in the tested honeys was calculated.

#### 2.2.2. Total Phenolic Content (TPC)

Total phenolic content analysis was conducted using the Folin–Ciocalteu assay [9]. In this procedure, 0.5 mL of the sample (10% water solution) was mixed with 2.5 mL of Folin–Ciocalteu reagent (0.2 N). Subsequently, 2 mL of sodium carbonate (75 g/L) was added after 5 min. The samples were then incubated for 2 h in the dark at room temperature. The absorbance was measured at 760 nm against a water blank using Shimadzu UV mini-1240 spectrophotometer (Kyoto, Japan). To quantify the TPC, a calibration curve was constructed using gallic acid standard (Sigma-Aldrich, St. Louis, MO, USA) at five concentration levels (0–300 mg/L), resulting in an R^2^ value of 0.9942. The total phenolic content of honey was expressed in mg of gallic acid equivalents (GAE)/100 g.

#### 2.2.3. Total Content of Phenolic Acids (TCPA)

The determination of phenolic acids followed the method outlined by Bueno-Costa et al. [6]. Specific aliquots from a honey solution (0.25 mL; 100 mg/mL in 50% ethanol), 0.25 mL of acidified ethanol solution (0.1% HCl in 95% ethanol) and 4.55 mL of a 2% HCl solution were transferred to a 10 mL volumetric flask. The mixture was homogenized and allowed to stand for 15 min. Subsequently, the absorbance was measured at 320 nm using Shimadzu UV mini-1240 spectrophotometer (Kyoto, Japan). The standard curve was established using caffeic acid (Sigma-Aldrich) at concentrations of 0 to 0.8 mg/mL (R^2^ = 0.9954). The results were expressed in mg of caffeic acid equivalents (CAE)/100 g.

#### 2.2.4. Determination of Free Radical Scavenging Activity by DPPH Method

To assess the ability of honey to scavenge the 1,1-diphenyl-2-picrylhydrazyl hydrate (DPPH) radical, we followed the method outlined by Wilczynska [7]. First, 2 g of samples were dissolved in 10 mL of distilled water, mixed and then filtered. Subsequently, 0.75 mL of the sample was combined with 2.25 mL of 0.1 mmol/L DPPH in methanol. Simultaneously, a control sample substituting distilled water for the honey was prepared. The solutions were mixed well and allowed to incubate (room temperature, protected from light) for 60 min. Absorbance was measured at 517 nm. The antioxidant capacity, represented as the percentage of DPPH inhibition (%AA_DPPH_), was calculated using the following equation:

(1) $$\%\mathrm{AA}=\frac{ABS\;control-ABS\;sample}{\begin{array}{c} ABS\;control \end{array}}\times 100$$

#### 2.2.5. ABTS Radical Scavenging Assay

The determination of the antioxidant activity of honey samples against the stable radical cation ABTS (2,2′-azinobis-[3-ethylbenzothiazol-6-sulphonic acid]) was carried out according to the method described by Wilczynska [7]. The ABTS was prepared by reacting a 7 mM ABTS solution with a 2.4 mM potassium persulfate solution. The resulting solution was stored in the dark for 24 h. The resulting ABTS solution was diluted with methanol until it reached an absorbance of 0.7 at 734 nm. Then, 6 mL of ABTS solution was added to 0.1 mL of the 20% sample solution. Everything was mixed thoroughly. After 15 min, absorbance was measured at 734 nm. Control samples were also prepared using distilled water instead of honey solution. The percentage inhibition was calculated as the ABTS radical scavenging capacity using Equation (1).

#### 2.2.6. Brown Pigment Formation

The evaluation of brown pigment formation (BPF) in the samples followed the procedure outlined by Turkmen et al. [19]. The determination of BPF involved measuring the absorbance of 4°Bx diluted extracts at 420 nm using Shimadzu UV mini-1240 spectrophotometer (Kyoto, Japan).

#### 2.2.7. Color Analysis

The Pfund classifier method was used to assess the color intensity of honey [20]. The samples underwent dilution to 50% with distilled water, then were thoroughly mixed and filtered. Subsequently, the absorbance was measured at 635 nm using Shimadzu UV mini-1240 spectrophotometer (Kyoto, Japan). The color intensity was then determined using the Pfund scale and calculated using the following equation:

Pfund = −38.70 + 371.39 × Abs [mm].(2)

#### 2.2.8. Melanoidin Content Estimation

The average absorbance of the honey samples was assessed using the procedure outlined by El Sohaimy et al. [21]. Samples were diluted to 50% (*w*/*v*) with warm (45–50 °C) distilled water, and the resultant solution underwent filtration with a 0.45 μm filter. Absorbance was recorded at two wavelengths: 450 nm and 720 nm. The difference in absorbance was quantified in milliabsorption units (mAU).

#### 2.2.9. Color Coordinates

The color coordinates of the samples were determined using the CIE Lab* method [2]. A Minolta chromameter (Model CR-400, Tokyo, Japan) was utilized for measuring the color of honey samples. The samples, with a weight of 20 g, were positioned in 20 mm thick holders and assessed against a black and white background at room temperature. CIE Lab* coordinates were recorded, where the L* value denotes the brightness of the honey (0 indicating dark, 100 indicating bright), a* represents the redness (+a) or greenness (−a) and b* signifies the degree of blueness (−b) or yellowness (+b).

#### 2.2.10. Analysis of Phenolic Compounds by HPLC

Gallic acid, chlorogenic acid, catechin, syringic acid, epigallocatechin gallate and *p*-coumaric Sigma-Aldrich (Sigma Aldrich) stock standard solutions (1 mg/mL) were prepared by dissolving them in methanol–water (80:20, *v*/*v*). In the extraction process, 1 g of honey sample was dissolved with 5 mL of methanol–acidified water (40:60, pH = 2, HCl, *v*/*v*) in a volumetric flask. The mixture was stirred for 15 min. The resulting extracts were filtered through nylon syringe filters (0.45 µm pore size). Chromatographic analysis was performed on a Shimadzu LC-40DXR Nexera system (Kyoto, Japan) equipped with a DAD detector. The separation was performed on a Phenomenex Kinetex C18 column (particle size 5 µm, 150 mm × 4.60 mm, 100 Å, Phenomenex, Torrance, CA, USA) using isocratic elution with an acetonitrile–0.1% acetic acid mixture (87:13, *v*/*v*). Oven temperature was −22 °C. Injection volume was 20 µL. Mobile phase flow was −1 mL/min. Wavelengths used were 275 and 306 nm. Retention times of chromatographic standards were used to identify phenolic compounds; however, the average peak areas and calibration curves of individual standards were used to quantify the content of individual compounds.

### 2.3. Statistical Analysis

STATISTICA 13.3 software (StatSoft, Inc., Tulsa, OK, USA) was used for statistical analysis. The one-way analysis of variance (ANOVA) method was used to determine correlations between the analyzed parameters. The Tukey test was used to assess the significance of differences between the mean values of the analyzed features (*p* = 0.05).

## 3. Results and Discussion

### 3.1. Water Content

Moisture is a characteristic of honey that is influenced by plant source, regional relative humidity and processing and storage conditions [3,21]. Reduced moisture content during honey storage can lead to caramelization and the Maillard reaction. Conversely, increased water content may induce honey fermentation and the production of acetic acid, both of which are undesirable [2]. The moisture content, as presented in Table 1, ranged from 15.6% to 18.9% in the investigated samples. The highest moisture contents were obtained in H2 and H4 samples, while the lowest was recorded for H7 samples. All analyzed honeys were characterized by moisture content below 20%, which is a desirable value in light of legal provisions relating to honey [1]. The obtained results align closely with those previously reported [22,23].

### 3.2. Total Phenolic Content

The total phenolic content of the tested honeys is presented in Table 1. In this method, the oxidation probe is reduced by antioxidants present in the honey (electron transfer occurs), resulting in the formation of a blue complex with maximum absorbance at 745–765 nm [24]. The results of the Folin–Ciocalteu reaction in honey must be interpreted as a quantitative estimate of the total phenolic content, as reducing sugars present in honey may also react with the FC reagent. However, since the proportion of sugars in different samples is constant, differences in FC test results reflect differences in phenolic content [25]. The content of phenolic compounds ranged from 16.90 mg GAE/100 g in acacia honey (H1) to 185.76 mg GAE/100 g in buckwheat honey (H6). The phenolic content of buckwheat honey was much higher than that of manuka honey and amounted to 66.49 mg GAE/100 g. Manuka honey and honeydew honey showed identical levels of TPC content. Similar relationships were noted by other researchers in previous studies [5,7,8,15]. The higher content of total polyphenols in buckwheat honey is due to the specific properties of the plant from which it comes. Buckwheat contains significant amounts of rutin, quercetin and other flavonoids, which are powerful antioxidants [26]. In addition, buckwheat flowers under specific environmental conditions that favor the accumulation of polyphenols in the nectar.

### 3.3. Total Content of Phenolic Acids

The total phenolic acid content of the honeys analyzed ranged from 3.57 to 18.83 mg CAE/100 g (Table 1). Buckwheat honey (H6) had the highest content of phenolic acids, while samples H1, H2, H3, H4 and H5 (light honeys) had the lowest. In the majority of the honeys tested, phenolic acids represented a low percentage of phenolic compounds, ranging from only 9% (H2) to 18% (H5). The content of TCPA in relation to TPC was higher in multifloral honey, in which phenolic acids accounted for 18% of the total content of phenolic compounds. These differences in the content of phenolic acids compared to the total phenolic content can be attributed to the non-specific nature of the spectrophotometric method used, as it detects all phenolic groups present in the solution (e.g., protein and ascorbic acid) [6].

### 3.4. DPPH and ABTS Assay

Two free radical scavenging methods (DPPH and ABTS) were used to determine the antioxidant activity of honeys. The DPPH method covers only some of the most reactive antioxidant components, while the ABTS method gives inconclusive results due to the presence of vitamins C and E and carotenoids, among others, in honey. Flavonoids, which react to form products with stronger antioxidant properties than the parent compounds, are also responsible for the inflated results [27].

DPPH, a stable nitrogen-centered radical, serves as a widely employed tool for evaluating the free radical scavenging capacity of diverse samples. The reduction capability of DPPH was determined by monitoring the decrease in its absorbance at 517 nm, induced by antioxidants. A positive outcome in the DPPH test signifies that the samples exhibit free radical scavenging properties [28]. The DPPH radical scavenging effects of honey samples are detailed in Table 1. The scavenging activity ranged from 21.2% in acacia honey (H1) to 80.7% in manuka honey (H8). The outcomes of the antioxidant properties of the examined honeys indicate that manuka honey demonstrated a higher antioxidant capacity compared to the Polish honeys, with honeydew honey exhibiting a similar antioxidant activity to manuka honey. Similar findings were reported by Socha et al. [5] and Gośliński et al. [15]. Wilczyńska [7], in an analysis of 10 distinct Polish honey samples, observed higher DPPH content and antioxidant activity in the following order: acacia < goldenrods < rape < lime < nectar-honeydew < multifloral < buckwheat < honeydew < phacelia < heather.

The ABTS activity was measured as the percentage of radical inhibition by antioxidant compounds present in honey. Significant variability in inhibition was observed between the tested samples. Buckwheat honey (H6) turned out to be the most effective radical scavenger, while acacia honey (H1) showed the lowest radical scavenging inhibition effect, as shown in Table 1. The increase in antioxidant capacity of the tested honeys occurred in the following order: H1 < H5 < H4 < H2 < H3 < H8 < H7 <H6. Manuka honey (H8) showed much lower antioxidant activity against ABTS radicals compared to buckwheat honey (H6) and honeydew honey (H7). Other researchers also reported consistent results [5,7].

### 3.5. Color

The data for the color parameters of the investigated honeys are presented in Table 2. The honey color was determined using several methods: brown pigment formation, the Pfund scale, which is able to give the color intensity of honey in the amber scale, melanoidin content and colorimetric parameters analysis in the CIELAB system. The analyzed honey samples of different floral origins possessed different color parameters.

The BPF (brown pigment formation) content ranged from 0.132 to 1.037. The highest value was determined in buckwheat honey (H6) and the lowest in acacia honey (H1). Manuka honey (H8) and honeydew honey (H7) demonstrated comparable BPF levels at 0.597 and 0.511, respectively.

Color is a vital sensory factor influencing consumer preference for honey quality. To classify honey colors, the Pfund scale is as follows: water white, extra white, white, extra light amber, light amber, amber and dark amber, with water white being the brightest and dark amber the darkest. Among the studied honeys, four color classes were identified: water white (25%), extra white (12.5%), white (50%) and light amber (12.5%). Light-colored honeys have a more delicate flavor, while dark honeys have a more distinctive flavor [6]. Markets in different countries around the world require specific colors of honey. For example, Germany, Austria and Switzerland prefer darker honey with a distinct flavor, while in North America, light honey with a more delicate flavor is preferred [18].

A key role in distinguishing the botanical origin of honey samples is played by the estimation of the content of compounds such as melanoidins and polymers that give the brown color [19]. Melanoidins are produced by combining sugars and amino acids. These processes occur at high temperatures and low water activity [29]. Consequently, the dark color of certain honeys can primarily be attributed to melanoidin formation, potentially indicating extended storage periods and/or honey-heating processes [30]. Furthermore, it is noteworthy that the color of honey is influenced by the content of polyphenolics as well as melanoidins [31]. Among the studied honeys, the highest melanoidin content was determined in buckwheat honey (H6) and the lowest in acacia honey (H1), respectively, 1203.3 mAU and 78.7 mAu.

In assessing the color of the honeys, the CIELAB Lab* color parameters are presented in Table 3. The L* values of the samples ranged from 39.1 to 71.8, indicating that all honeys were relatively bright or pale. Acacia honey (H1) exhibited the highest luminosity level. Low a* values in honey samples suggest minimal red coloration in these honey types. The negative a* values indicate the presence of some green components, observed in the case of rape (H4) and acacia (H1) honey. Manuka honey (H8) showed the highest yellowness values (high b* value), while buckwheat honey (H6) was identified as having the greatest blueness (negative b* value). Consistent findings have been reported by other researchers [2,4,7,16,32]. Can et al. [11] observed that the color of honey depends mainly on the origin of the nectar and the pollen content of the honey. Both of these ingredients contain various color pigments, i.e., anthocyanins, phenolic acids, proanthocyanins, flavonoids and minerals. Additionally, Kavanagh et al. [33] found that the color of honey may also be influenced by the practices used by beekeepers, in particular, the frequency of wax replacement. Moreover, external factors such as contact with metals, high temperatures and light can also affect the color of honey.

### 3.6. Analysis of Phenolic Compounds by HPLC

Phenols found in honey are mainly benzoic and cinnamic acid esters, as well as flavonoids. The phenolic profile in individual honeys varies and depends primarily on its botanical origin because these compounds come mainly from nectar, pollen and propolis [29,34]. The type and amount of phenolic compounds may also vary depending on the season, the climatic conditions and the processing used [35]. The analyzed samples also contained other substances that were characterized by similar phenolic spectra and similar chromatographic behavior. Unfortunately, they could not be identified due to the lack of availability of patterns. The phenolic profiles of the tested honey samples are presented in Table 4. Polyphenols found in all the honeys tested are gallic acid and catechin. A high concentration of gallic acid (296.13 μg/g) was detected in buckwheat honey (H6), while the lowest value was identified in acacia honey (H1, 96.15 μg/g). The highest catechin content was obtained in the lime honey (H3, 71.63 μg/g) and the lowest in acacia honey (H1, 9.02 μg/g). Chlorogenic acid was only detected in H1, H3, H4, H5 and H7 at 2.06, 23.69, 4.84, 14.48 and 4.10 μg/g, respectively. Syringic acid was determined only in lime honey (H3, 3.19 μg/g). Epigallocatechin gallate was detected in H3, H5, H6 and H7 at 4.00, 9.67, 5.33 and 7.33 μg/g, respectively. H3 had the highest values of p-coumaric acid (17.63 μg/g), and H2 had the lowest values (1.36 μg/g) among the honey samples. The presence of large amounts of syringic acid in lime honey has also been determined by Puścion-Jakubik et al. [17].

### 3.7. Correlations

In order to identify correlations between the parameters studied, Spearman’s correlation coefficients were used, which are a measure of the strength of the relationship between the variables studied. The closer the coefficient is to 1 or −1, the stronger the relationship between the variables. The correlations established between the total phenolic content, total phenolic acid content, antioxidant capacity, color parameters and phenolic compounds are presented in Table 5, showing the relationships between these values. The analysis revealed that the total phenolic content is strongly correlated with antioxidant activity AA_ABTS_ (0.97). Additionally, strong correlations were also noted between total phenolic acid content and catechin (0.97), antioxidant activity AA_DPPH_ and a* (0.99), brown pigments and L* (0.99), brown pigments and b* (0.99), brown pigments and melanoidins (0.94), brown pigment and syringic acid (1.00), L* and b* (0.98), L* and syringic acid (0.99) as well as b* and syringic acid (0.99), chlorogenic acid and catechin (0.94) and chlorogenic acid and *p*-coumaric acid (0.94). Conversely, negative correlations were stated between total phenolic content and a* (−0.99), total phenolic content and AA_DPPH_ (−0.99), total phenolic acid content and AA_DPPH_ (−0.94), total phenolic acid content and a* (−0.95), AA_DPPH_ and AA_ABTS_ (−0.94), brown pigments and melanoidins (−0.94), melanoidin and L* (−0.96), melanoidins and syringic acid (−0.94), as well as between gallic acid and *p*-coumaric acid (−0.98). Antioxidant activity AA_ABTS_ was negatively correlated with BFP, Pfund scale, L*, a*, b* and syringic acid (−0.96, −0.98, −0.97, −0.93, −0.99 and −0.96, respectively).

The strongest correlation was noted between color parameters (melanoidins and brown pigments) and total phenolic content, indicating that the darker honey can be attributed to the presence of the high amount of phenolics, thereby enhancing its antioxidant capacity. For instance, buckwheat honey (H6) exhibited the highest average color value among the samples, indicating elevated total phenolic content (185.76 mg GAE/100 g) and phenolic acids (18.83 mg CAE/100 g). The strongest correlation between antioxidant activity AA_ABTS_ and phenolics confirms that phenolics are the major contributors to the radical scavenging activity in honey samples. Similar correlations were reported in previous studies, such as between color and phenolics (0.974) by Al-Farsi et al. [20]; antioxidant and phenolics (0.962) by Bouhlali et al. [36]; and color and phenolics (0.816), color and antioxidants (0.820) and phenolics and antioxidants (0.785) by Moniruzzaman et al. [37]. Pontis et al. [38] also reported a high correlation between color and phenolics (0.967), color and antioxidants (−0.800), as well as phenolics and antioxidants (0.785). Budzynski and Miotto [39] suggested that phenolics in honey may be components of the melanoidin structure, indicating a direct interaction between polyphenols and melanoidins that influences the function of melanoidins, booth their loss and gain. Furthermore, Imtara et al. [40] obtained a strong positive correlation between color on one side and melanoidin and polyphenols. Many authors have consistently established a significant relationship between phenol content and the antioxidant activity of honey, highlighting the pivotal role of phenols in scavenging activity [41,42,43].

## 4. Conclusions

Analysis of the content of total phenolics, total phenolic acids, antioxidant activity and color parameters in selected samples of Polish and manuka honeys showed significant differences in the above parameters between the different types of honey. The high polyphenol content in buckwheat honey gives it stronger antioxidant properties, making it more valuable from a health point of view. In terms of color, manuka honey was similar to honeydew honey, but both were lighter than buckwheat honey, which had the highest color parameters. Strong correlations were found between phenolic content, antioxidant activity and color, highlighting that darker-colored honeys such as buckwheat, honeydew and manuka have higher phenolic content and, therefore, stronger antioxidant activity. Chromatographic analysis revealed differences in the content of selected phenolic compounds in the honeys tested. For example, syringic acid was only detected in linden honey, suggesting that the presence of this compound can be used to identify this type of honey. Honeydew honey was the only one of all the honeys tested in which *p*-coumaric acid was not detected, which could also be a characteristic feature used to identify the botanical origin of this honey. The study undoubtedly deepens our understanding of honey diversity and highlights the potential importance of regional honey varieties in promoting health-enhancing properties. In light of the results obtained, we suggest further research and in-depth study of Polish buckwheat honey, promoting its increased consumption due to its significant nutritional values and exceptional bioactive properties.

## Figures and Tables

**Table 1:** The content of water, total phenolic (TPC), total phenolic acids (TCPA) and antioxidant activity (DPPH and ABTS) in analyzed honeys.

	Floral Sources	Moisture (%)	TPC (mg GAE/100 g)	TCPA (mg CAE/100 g)	AA_DPPH_ (%)	AA_ABTS_ (%)
H1	*Robinia pseudoacacia*	17.8 ^d^ ± 0.0	16.90 ^a^ ± 0.30	4.29 ^a^ ± 0.00	21.2 ^a^ ± 1.8	5.4 ^a^ ± 1.7
H2	*Phacelia tanacetifolia* Benth.	18.9 ^e^ ± 0.2	39.96 ^e^ ± 0.66	3.83 ^a^ ± 0.02	50.0 ^c^ ± 1.4	16.1 ^cd^ ± 1.2
H3	*Tilia*	17.3 ^c^ ± 0.2	33.24 ^d^ ± 0.30	3.57 ^a^ ± 0.04	50.0 ^c^ ± 2.4	18.6 ^d^ ± 0.6
H4	*Brassica napus*	18.9 ^e^ ± 0.2	29.76 ^c^ ± 0.16	3.83 ^a^ ± 0.03	36.2 ^b^ ± 1.2	12.9 ^bc^ ± 0.9
H5	*Multifloral*	18.6 ^e^ ± 0.0	26.21 ^b^ ± 0.22	4.86 ^a^ ± 0.06	32.1 ^b^ ± 1.4	11.9 ^b^ ± 1.8
H6	*Fagopyrum esculetum*	16.5 ^b^ ± 0.2	185.76 ^g^ ± 0.90	18.83 ^d^ ± 0.36	49.4 ^c^ ± 1.5	71.8 ^g^ ± 1.2
H7	*Honeydew honey*	15.6 ^a^ ± 0.2	66.07 ^f^ ± 0.80	9.03 ^c^ ± 0.06	80.0 ^d^ ± 0.9	55.6 ^f^ ± 1.6
H8	*Leptospermum scoparium*	18.6 ^e^ ± 0.0	66.49 ^f^ ± 0.52	7.31 ^b^ ± 0.26	80.7 ^d^ ± 2.2	38.4 ^e^ ± 1.8

Different letters in the same column indicate significant differences between means at the 5% level (*p* < 0.05).

**Table 2:** The characteristics of honey color.

	BPF	Pfund Scale (mm)	Color Description	Melanoidins (mAU)
H1	0.132 ^a^ ± 0.002	−5.7 ^a^ ± 0.4	Water white	78.7 ^a^ ± 2.5
H2	0.239 ^b^ ± 0.001	2.5 ^b^ ± 0.4	Water white	180.7 ^b^ ± 1.5
H3	0.280 ^c^ ± 0.001	11.7 ^c^ ± 0.2	Extra white	218.0 ^c^ ± 2.6
H4	0.358 ^e^ ± 0.002	33.8 ^f^ ± 0.2	White	241.3 ^d^ ± 6.7
H5	0.305 ^d^ ± 0.002	19.5 ^d^ ± 0.4	White	268.7 ^e^ ± 2.9
H6	1.037 ^h^ ± 0.002	69.0 ^g^ ± 0.4	Light amber	1206.3 ^h^± 4.7
H7	0.511 ^f^ ± 0.001	33.1 ^f^ ± 0.2	White	487.3 ^f^ ± 1.2
H8	0.597 ^g^ ± 0.001	29.1 ^e^ ± 0.2	White	520.3 ^g^ ± 3.1

Different letters in the same column indicate significant differences between means at the 5% level (*p* < 0.05).

**Table 3:** The color coordinates of the sample honeys.

	L*	a*	b*
H1	71.8 ^e^ ± 0.3	−1.5 ^c^ ± 0.0	14.4 ^d^ ± 0.2
H2	55.0 ^b^ ± 0.3	6.1 ^f^ ± 0.1	15.7 ^e^ ± 0.4
H3	52.0 ^a^ ± 2.1	3.1 ^e^ ± 0.1	18.5 ^f^ ± 1.7
H4	46.5 ^c^ ± 1.0	−2.5 ^b^ ± 0.1	13.0 ^c^ ± 0.1
H5	53.7 ^ab^ ± 0.8	1.7 ^a^ ± 0.0	7.2 ^a^ ± 0.5
H6	39.1 ^d^ ± 0.6	1.8 ^a^ ± 0.2	−2.6 ^b^ ± 0.3
H7	44.8 ^c^ ± 0.3	11.2 ^g^ ± 0.3	6.6 ^a^ ± 0.3
H8	52.9 ^ab^ ± 1.1	2.3 ^d^ ± 0.2	23.9 ^g^ ± 0.6

Different letters in the same column indicate significant differences between means at the 5% level (*p* < 0.05).

**Table 4:** Phenolic compounds in honey sample [μg/g].

	Gallic Acid	Chlorogenic Acid	Catechin	Syringic Acid	Epigallocatechin Gallate	*p*-Coumaric Acid
H1	96.15 ± 0.02	2.06 ± 0.07	9.02 ± 0.05	ND	ND	2.02 ± 0.01
H2	144.06 ± 0.01	ND *	18.34 ± 0.11	ND	ND	1.36 ± 0.11
H3	122.52 ± 0.01	23.69 ± 0.21	71.63 ± 0.41	3.19 ± 0.13	4.00 ± 0.65	17.63 ± 0.21
H4	128.31 ± 0.02	4.84 ± 0.21	15.40 ± 0.51	ND	ND	2.40 ± 0.31
H5	167.57 ± 0.21	14.48 ± 0.43	11.08 ± 0.03	ND	9.67 ± 0.89	4.81 ± 0.05
H6	296.13 ± 0.02	ND	68.00 ± 0.37	ND	5.33 ± 0.73	1.40 ± 0.07
H7	276.52 ± 0.01	4.10 ± 0.21	16.10 ± 0.31	ND	7.33 ± 0.16	ND
H8	186.06 ± 0.01	ND	25.14 ± 0.21	ND	ND	8.41 ± 0.61

* ND—not detected.

**Table 5:** The Spearman’s correlation coefficients among total phenolic content, total phenolic acid content, antioxidant activity, color and phenolic compounds.

	TPC	TCPA	AA_DPPH_	AA_ABTS_	BFP	Pfund Scale	Melanoidins	L*	a*	b*	Gallic Acid	Chlorogenic Acid	Catechin	Syringic Acid	Epigallocatechin Gallate	*p*-Coumaric Acid
TPC	1															
TCPA	0.89	1														
AA_DPPH_	−0.99	−0.94	1													
AA_ABTS_	0.97	0.76	−0.94	1												
BPF	−0.87	−0.55	0.80	−0.96	1											
Pfund Scale	−0.33	−0.72	0.44	−0.98	−0.18	1										
Melanoidins	0.66	0.25	−0.56	0.81	0.94	0.50	1									
L*	−0.84	−0.50	0.76	−0.97	0.99	−0.24	−0.96	1								
a*	−0.99	−0.95	0.99	−0.93	0.79	0.46	−0.54	0.75	1							
b*	−0.92	−0.66	0.87	−0.99	0.99	−0.05	−0.89	0.98	0.86	1						
Gallic Acid	0.00	−0.44	0.12	0.24	−0.50	0.94	0.76	−0.55	0.14	−0.38	1					
Chlorogenic Acid	0.50	0.83	−0.60	0.28	0.00	−0.98	−0.33	0.06	−0.62	−0.13	−0.87	1				
Catechin	0.76	0.97	−0.83	0.58	−0.33	−0.87	0.00	−0.27	−0.84	−0.45	−0.66	0.94	1			
Syringic Acid	−0.87	−0.55	0.80	−0.96	1.00	−0.18	−0.94	0.99	0.79	0.99	−0.50	0.00	−0.33	1		
Epigallocate-Chin Gallate	0.00	0.44	−0.12	−0.24	0.50	−0.94	−0.76	0.55	−0.14	0.38	−1.00	0.87	0.66	0.50	1	
*p*-Coumaric Acid	0.19	0.60	−0.31	−0.05	0.33	−0.99	−0.62	0.38	−0.32	0.19	−0.98	0.94	0.79	0.33	0.98	1

## Data Availability

The original contributions presented in the study are included in the article, further inquiries can be directed to the corresponding author.
